# The Story of White Coats and Small Miracles

**DOI:** 10.5334/cie.4

**Published:** 2020-01-09

**Authors:** Magdalena Picz

**Affiliations:** 1 Independent author, PL

**Keywords:** Cancer, life story, young girl, teenager, coping, development

## Abstract

This is the story of a girl who refused to give up, no matter what. The words tell the story of sadness, bravery, loss, sacrifice, and pain, but also of enormous happiness, devotion, and the realization that the extraordinary is to be found in the smallest things we do every day.

*My name is Magda and I’m 21 years old. I was a patient many times and now I try to see how it is on the other side by studying nursing at the University of Medical Sciences in Poland. I’m also a volunteer in one of the oncology departments. I have a younger sister, who always makes me laugh, and my mother, who is with me whenever I need her. I love reading, traveling, learning new things and writing from time to time. I try to help a little and do some good whenever I have the chance, believing that nothing is impossible*.

*This story took me back to the very beginning of my life. I never imagined it would be that difficult but I’ve decided to write it because if there is anyone I can help in that way, I want to do it*.

My name is Magdalena, but my friends and family call me Madzia. I’m 21 years old. I was a patient many times, and now I want to help others as much as my doctors, nurses, therapists and teachers helped me 20 years ago. That is why I decided to study nursing at the University of Medical Sciences in Poznań, Poland. I’m also a volunteer in one of the oncology departments.

I have a younger sister, who always makes me laugh, and my mother, who is with me whenever I need her. I love reading, travelling, learning new things and writing from time to time. I try to help a little and do some good whenever I have the chance. When I was five years old, my mother told me, “Nothing is impossible.” I have never heard anything closer to the truth, but when I first heard it I thought it was a lie. To me, people who thought like this were naïve. I was sure some things in life were impossible to achieve and there was nothing we could do about it, but then I survived the hardest times in my life, fulfilled some of my biggest dreams, gave a speech at the 11^th^ HOPE Congress in 2018 in Poznań and I thought to myself that maybe nothing really is impossible after all.

This is probably the most private thing I have ever written, but I decided to share it because there may be a chance it will help somebody some day and, in the process, make “the implicit explicit and the hidden seen” (Atkinson, 1998, p. 125). The following words tell the story of how I became who I am now. In the end, everyone decides on their own who they are, but some aspect of it will reflect people they have met and the experiences they have had to endure. At a very young age I had to develop certain personality traits that helped me survive. Luckily, I had people with kind hearts and brave spirits to learn from. It has been a long journey, and I decided I’d remember the best of it. My friends and family have always been the best part of me.

In October 1988, in a small village in the centre of Poland, a girl named Hannah went to a party where she fell in love with a tall, handsome boy with dark, curly hair, who was wearing a dark leather jacket and faded blue jeans. He asked her to dance with him. They spent hours together, laughing and chatting, and then he offered to drive her home. When he asked where she lived, Hannah smiled and said, “You will find me, if you want to.” He went to look for her first thing in the morning the next day. Ten years later, in 1998, on Tuesday, April 28, in Poznań, Poland, their baby daughter came into this world.

When I close my eyes and try to think back to my earliest memories, I am two years old, and all I see are white coats of medical students standing behind the backs of their professors. I see sparkles shining shyly in their eyes as they listen carefully to their mentors and a barely visible shadow of fear of what the future holds for them. Somewhere in the corner nurses watch closely while taking notes. They are all talking in some weird language I don’t understand, but the voice of the doctor is so calming that I don’t care. My mom is waiting in the hallway of Karol Jonscher Clinical Hospital in Poznań. She looks worried, her hands are shaking slightly, and she seems to be in a different place. I catch the sight of a young female student and smile. She smiles back, and for a fleeting moment, I feel a bit better. The professor closes a dark, green folder he’s holding. Today’s round is over. I watch them leave my room, and suddenly I feel extremely sad. They seem so nice; I want to play with them. One of the nurses stops and smiles at me for the last time before she shuts the door. “I hate hospitals,” I think to myself, but then the door opens again and my mom walks in. Great, now we’re going to read some books!

I hardly remember anything from my time in the oncology department. I know it from my mother’s stories, but this is what I recall from my own memory.

## Fairytale in a False Mirror

The story of how my parents met seemed to be almost like a fairytale to me. My mother was a dressmaker, who loved partying, adventures, hanging out with friends; he was a car mechanic and also a singer in a band he and his brothers created. He would often come and take her on long car rides where they wouldn’t be bothered by anyone. I imagine they must have felt like they could take over the world anytime because it couldn’t exist without them.

After many unsuccessful attempts, my mother got pregnant. My father promised himself he would stop smoking if I was born healthy, and he kept that promise. I was born healthy. I was in perfect shape; I even got 10 points on the Apgar scale. My mom once told me how overjoyed she was when she heard this. My health was the only thing that mattered to her.

The fairytale ended when I fell ill seven months after my birth.

In November 1998, when I was seven months old, I started crying in the middle of the night for no particular reason. My mom tried to calm me down until I fell asleep. Exactly an hour later, I woke up and started crying and screaming again. The same scenario repeated itself every single night. Worried, scared to death and alone, my mom visited many different doctors in three cities with me. They all made different diagnoses and then prescribed some painkillers – all to no avail. Clearly, I was in pain, but my mom had no idea why, and it was slowly killing her inside but she had no other choice but to wait, hoping for better days to come.

Other problems awaited her at home. My father was rarely at home. He often shouted a lot; however, there were times when he acted like the best dad ever. We played video games – like adventure games and car races – together. As I grew older, I remember my friends telling me how funny and awesome my dad was. “I wish I had a dad like yours,” I’d often hear them say.

A few weeks and a couple of different doctors later, my mom found another general practitioner. He prescribed some antibiotics, painkillers and other medications for me. When nothing helped, we came back to him two weeks later. “Listen, I have no idea what’s going on. You should go to a pediatric clinic, she ought to be seen by a specialist,” he said and that was the best advice my mother could have heard. Among all those medical practitioners we met, in my opinion, he was the wisest. He did one of the hardest things to do – which was admitting he didn’t know what was wrong – and was one of the first people who contributed to saving my life.

My mom felt lost. Both my mother’s and my father’s families were religious, so my mom often found herself kneeling on a marble floor in an enormous basilica in my home town. She spent hours asking God what she should do to keep me safe and alive because she had no one else to ask. Ironically, back then that cold marble floor was the warmest place on earth. One chilly afternoon she got up, took one last look at the church walls, which had been holding her together somehow, and she decided to go to the clinic in Poznań with me.

On November 25, as I was standing, with my mom holding me by the hands in our backyard, all of a sudden I fell to the ground. That was the first episode of the paresis of my lower limbs. I was unable to move my legs for weeks after that.

## Possibly the Worst Christmas Ever

About a week later, on December 2, the glass doors of the pediatric clinic in Poznań opened, and we were welcomed by a quiet rustling of white coats. I was eight months old and it was my first hospitalization. We spent a lot of time on different wards; I was examined by many practitioners, who suspected many different diseases and ran lots of tests, which leading to the conclusion that I had a neurological disease.

Three days later, I was moved to the department of infectious diseases in the same clinic, with bronchitis. During the treatment, the doctors noticed the paresis and the lack of some physical neurological reflexes. They performed a spine biopsy on me.

Finally, on December 23, the neurosurgeon told my mom what they discovered as a result of all the tests they had run. Only one day before Christmas Eve in 1998 my mom found out I had cancer.

It had a weird name – *neuroblastoma*; a tumor right in the centre of my spinal cord; a bunch of incorrect cells in the shape of an hourglass. My doctors made the decision to perform two surgeries, so on December 28 the nurses took me to the operating theatre. The surgery took eight hours, during which my mom was waiting outside the heavy doors of the operating theatre while talking to one of my doctors about it all. He was trying to calm her down. He didn’t quite succeed, but knowing my mom, I’d say he deserved a gold medal for even trying.

The second surgery was performed a few weeks later, January 8. Shortly afterwards, I went to the oncology ward in the same pediatric clinic to undergo chemotherapy for the next 14 months. I was nine months old.

## Late-Night Conversations in a Hospital Coffee Bar

Twenty years ago, parents were not allowed to stay with their children in the hospital during the night. The oncology department in Poznań opened at 10 A.M. and closed at 8 P.M.

I was bald, pale, I had no eyebrows and I didn’t like what I saw each time I looked in the mirror. I didn’t like anyone besides my mom and the medical students to see me. On January 18, I got my fist dose of chemotherapy. On that day a nurse, alarmed by the sound of a baby crying, came to my room. My face was red, and I was burning up. My mother had gone to the toilet a few minutes before, and when she came back the nurse was furious and asked her why she had brought a child with fever to the hospital to get chemo. I didn’t have a fever. I was in that state because I was so frightened when I didn’t see my mom. She was my anchor. When she took me in her arms to hug me, I immediately felt safe, my breathing stabilized and the temperature dropped.

That evening, my mom was overcome by tiredness, and decided to get some coffee from the hospital coffee bar outside the main building when I fell asleep. I imagine she must have ordered black coffee to stay awake. As she sat drinking her coffee, her mind racing with a host of thoughts, suddenly the sound of a male voice, directed at somebody else, drew her attention, “You remember that little girl with a tumor in the spinal cord? I thought she was a lost cause. She had surgery at the last minute. If he had waited even a day, she would have died.”

She told me about that conversation many years later – when I was 16 and she was sure I was safe and healthy.

## A Better World

When something bad happens to children and they can’t do much about it, they will do anything to survive. At the age of two, my way of surviving was living in two parallel realities.

I truly loved books. Whenever I spotted a book, I immediately grabbed it and told my mom to read it to me. I was always so excited to hear about dark, mysterious castles, evil queens and breathtaking, dangerous adventures. I have a pretty good memory, so after hearing a story once I was able to repeat it from memory. When she was tired, my mom often would skip some words, pages even, but I immediately caught her and asked her to read it properly. I was a horrible kid during that period of my life, I won’t deny it.

When I think back and wonder what attracted me to the books that much, I realize that it was because the worlds in books were different – better than mine, for sure. And living a different life even for minutes only – even if it wasn’t real – was a thousand times better than being stuck in this hospital reality.

## “Pack Our Bags, We Are Going Home”

How does a mother spend weeks in hospital with her seriously ill child without going crazy? She walks, she thinks, she talks to other parents. What does she talk to them about? It’s simple. She talks about the only subject she feels almost like she was an expert on – illnesses. When I was asleep, my mother often spent her free time talking to other parents about their children’s diseases. Not only did she talk about them, but she also observed them. She saw how the children were slowly getting better. Sometimes she compared their improvements to mine, but sadly I didn’t improve, at least not until the very last month of my chemotherapy – when I was almost two years old. On February 16, 2000, my mom opened the door to my room and saw me standing on my bed leaning on the railing and looking her straight in the eyes with a smile on my bald, pale face that said “Mom, pack our bags, we are going home.”

I think a lot of medical practitioners working in the ward were happy I was going home because I was an extremely difficult patient. I associated everything – and almost every person working in the medical field with pain – and therefore, wasn’t willing to cooperate with my caregivers, afraid of feeling that pain again. I felt alone and misunderstood, and I think I needed someone to notice my fear and understand it.

On the last day of my chemo treatments, the head of the oncology department came to my room to give me a goodbye gift. She gave me three toy sheep – a big, a medium-sized, a small – each with a small gold bell around its neck. They were truly beautiful and very soft, but I was furious because, to me, this gift meant sympathy and I didn’t want that. I just wanted to go home – so when the doctor started to leave, I threw all three sheep at her.

Now she always smiles and hugs me whenever I come to the ward as a volunteer.

## Vague Memories

Slowly, my feet started to deform with time. I wore orthotics and orthopedic shoes. When I was 2, the deformation was so serious that I was unable to walk. It was a progressive process.

Besides my earliest memory, I remember one more thing about the hospital. In April 2001, a little over a year after I was dismissed from the hospital – when I was three years old – an orthopedic surgery temporarily straightened my feet. A few months later, my mother and I went to the oncology department to tell my doctors that the surgery had been successful. My mother opened the door for me, and suddenly I felt unimaginable pain, fear, anger and sadness and refused to go inside. The doctors had to come to me.

When I was five years old my feet were very deformed, but I didn’t care. I was happy and the shape of my feet or the way I walked never made me sad or angry. I liked myself, and I absolutely loved reading, paper dolls and pretending to be one of the characters from books and films. Long chilly, magical winter evenings with a cup of hot chocolate and my grandpa reading stories to me by my side are the warmest memories I have of my childhood.

I have had two surgeries to temporarily straighten my feet when I was three and nine years old, but I knew that when I reached maturity, I would have to undergo more surgeries to straighten my feet permanently.

My parents divorced when I was 11 years old. I was still a kid, but I understood everything. I knew it was coming, it was inevitable. For years, I had been watching my parents’ relationship slowly falling apart, piece by piece. I was often awakened by screams in the middle of the night. I knew they were fighting. I tried to go back to sleep, but it didn’t work, so I took my pillow, sat by the door and listened. After the divorce, I lived with my mother and my sister, who was born when I was 8 years old. The day she was born was one of the best days of my life. Now I occasionally talk to my father on the phone, but that’s it.

At the age of 12 years, I started to lower my head a little bit more, hide my face behind my long, dark hair a little bit more often, look down at my feet while walking, talk a little bit less and feel a little bit worse. It was like I was slowly fading away day by day.

At 13, I started going to a new school, and I remember being excited about it. New school meant new classes, which meant new people and opportunities. I was about to go to a place where no one knew anything about me, which was probably the best thing that could have happened to me because the place where I came from wasn’t good. I was bullied terribly, and I still remember almost every single time someone made fun of me or the way I walked.

On September 2, 2011, the first day at my new school, I woke up at 6 A.M. as usual. I opened the window in my room and heard the birds chirping. The wind was chilly, but the sun was shining so I thought it might be a really good day. I brushed my teeth, got dressed, ate breakfast, drank tea, took my bag and went to the bus station. Twenty minutes later the bus stopped at my new school. I noticed a group of students by the gate when I was getting off the bus. I never suspected that they would stand in my way to prevent me from walking any further, make fun of me while trying to take my bag from me and then spit at my shoes.

Looking back, right now, I still clearly remember this day… all the days after this are just a blur.

I spent three long years in that school. During this period of my life, every day was practically the same. I woke up feeling more exhausted than ever. I struggled to even open my eyes. I didn’t eat much; I didn’t care much about anything. I just didn’t see the point. I went through the motions every morning, took my bag and went to the bus station and then got off the bus only to see those same students waiting for me to make fun of me again, push me and take my bag. Unfortunately, that group of students grew with time. I always made sure not to be alone during breaks between lessons because I was too afraid to walk outside alone. Everywhere I went, whatever I did, I always had the feeling that nothing made sense anymore.

That is how my life looked back then. I thought it couldn’t get any worse. But it did!

It was a beautiful, spring day. I was 13 years old and in the first year of middle school. I was sitting on a bench in school courtyard during the last break of the day. Unfortunately, I was alone. My best friend was sick and couldn’t come to school. I felt a warm wind gently brushing my skin. I was enjoying a quiet moment when the bell rang. As I started walking towards the main entrance, I automatically quickened my pace the moment I heard familiar voices behind me. Suddenly I felt a hard stone hitting the back of my head.

I didn’t think much. I just wanted to get out of there and never come back, and that was what I did. I took my bag, I went to the restroom for a couple of minutes to recover a bit, and then I got out. The following month I lied to my mom and grandparents every day. Every single morning, I went to catch the bus but I didn’t go to school. I got out on the main station and I wandered about town for hours until 3 P.M., the time when my lessons usually ended. Then I went home, and when my grandma asked how school was going, I’d answer, “Great.”

The following autumn, October of 2012, I coughed in class one day, and noticed drops of blood on my palm. The doctors performed a series of tests, and I had a gastroscopy. It turned out I had bacteria in my stomach and duodenum, which could be caused by stress. No one knew how stressed I was, because I didn’t say anything, and no one asked. I felt hopeless because at school, everyone laughed at me every single day and yet, no one noticed anything. When I was in the second year of middle school, my friend suggested we tell the school counselor about the bullying, and so we did. In response, I was told that I didn’t have any proof and I shouldn’t accuse other students of such things. After that, I completely gave up trying to change the situation. I needed for someone to notice me and what was going on around me: I didn’t have any more strength to fight for myself all alone.

For the next month I stayed home from school. I would lie on my bed and stare blankly at the ceiling or sleep. I didn’t eat much, I didn’t talk to anybody; I didn’t do anything in particular. The only times I got out of bed was when I needed to go to the toilet. My mom was worried and scared to death; it was all visible in her eyes.

December was coming up, my favorite month, so I managed to get up from my bed and go to school to cheer my mom and grandparents up a bit, but my behavior didn’t change much. I wasn’t happy, I wasn’t sad – I just existed. Later I learned those were episodes of depression.

Later that month, my homeroom teacher came up to me during the lunch break one day. He said he wanted to talk to me about something, so we went to an empty classroom and sat at a table opposite each other. “I see what’s going on. It’s slowly killing you. You’re a young, smart and beautiful girl. Everything is fine with you. I’ve been a teacher for a long time, believe me, I know how they think and they won’t stop until you raise your head and speak up. I’m not an expert, but I think once you can see something beautiful in yourself, others will see it, too.” I never fully realized how much I needed that conversation. Finally, someone noticed me, took matters in his own hands and told me what I should do instead of focusing just on the situation. I didn’t do anything about it then because I wasn’t willing to fight for myself anymore; I didn’t see the point.

## Magnificent Transformation

I entered the almost empty orthopedic clinic on January 2, 2013. I was still mentally tired. After the necessary formalities, the department nurse showed me to my room. My mom was talking to me, but I couldn’t focus on what she was saying. Suddenly, there was a knock on the half-open door. I turned and saw a man with a wide smile on his face and bandages soaked in blood around his head. Worried, I asked what had happened and if he was alright. He laughed, ensured me everything was fine and proceeded to sit down on my bed. It was the first time I truly smiled in a long while. It turned out that he was the occupational therapist, and he made my time at the hospital not nearly as bad as I had expected it to be.

I had five surgeries in a year. During my time in the orthopedic clinic, I met a few hospital teachers. The chemistry teacher was my absolute favorite. I felt so comfortable with her. I wasn’t afraid to make mistakes and ask questions, even the ones I might otherwise have considered stupid. She was always consistent, but she understood whenever I felt down or sad or was just tired and unable to focus. She even gave me an A+ once, which made me feel like I was on top of the world for a minute because chemistry wasn’t really my cup of tea. Others might say getting an A+ in chemistry isn’t a big deal, but to me it was because I was depressed, I felt like I was stupid, I felt myself slowly fading away, almost like a shadow, and nothing made sense to me anymore, but this teacher had the ability to conduct our lessons in such a way that I understood everything. It showed me that maybe – just maybe – I wasn’t that stupid after all, and it awakened my love for learning. After our lessons, I had that barely perceptible feeling that maybe there is still something that could make me happy.

There were only two weeks during that whole year when I didn’t have a cast on my legs. I was lucky because my doctors allowed me to go to a language camp in London arranged by my school, provided that I rest whenever possible. One day we travelled on the subway train. There was only one seat that wasn’t taken. The last thing I wanted was to sit there because that seat was surrounded by the kids who had been making fun of me at school; however, my teacher insisted on it so I sat there, right in between those boys. I was terrified so I lowered my head, took a deep breath and waited for the inevitable. Little did I expect what happened next.

One of them spoke to me, and asked me what had happened to me and why my feet were like that. I told them. I told them everything, and from that moment we became friends and I realized one thing – they weren’t evil. They just didn’t know what they were laughing at. In fact, I think they are among the wisest people I have ever met because they understood their mistakes and that required a lot of wisdom and bravery. The remaining days of this camp were more than awesome. Those two weeks were a period of magnificent transformation for them and for me.

We didn’t talk anymore after that, but for a couple of days we were best friends and it was enough.

I admit it is hard for me to say what I would do if I had been in the teachers’ place. My role isn’t to judge them. I just think it would have been a huge help for me if they had been with me, paid more attention to my feelings and tried to encourage me to raise my voice to defend myself and grow stronger, like my homeroom teacher.

Each of my feet was deformed in a different way. I first had surgery on my right foot. I’d never imagined it would hurt that much, but afterwards, I was able to move my feet up and down which I hadn’t been able to do before. After this surgery, I woke up late in the evening with wires in my foot, so much pain but a dash of hope.

The second surgery, on my left foot, was a thousand times more complicated than the previous one. It is extremely hard to explain what it involved, but try to imagine a foot twisted like a spring. It didn’t look exactly like that but similar. All I wanted was to have normally looking feet, but I always thought it was out of my reach. But when I woke up after the second surgery and saw a normal shape of the plaster on my left foot, it was the moment when my life changed. Forever.

I had individual home teaching for a year after those two surgeries. The teachers were my main connection to my classmates. I felt this cozy warmth in my heart every time they told me that my friends at school missed me and wanted to know if I was okay. Receiving individual schooling allowed me to continue my education, and since I loved learning it made me happy – both at my home and in the hospital. I wasn’t afraid to ask questions when I didn’t understand something because there was only me and the teacher, so the process of learning was more effective. And also, I didn’t feel left out when I finally met my classmates again. I came back to school on the very last day of the school year with a smile on my face, my back straight, my hair cut and wearing a new red dress. I passed two students in the hallway and heard them say, “Who was that? Does she even go to this school?”

Before I was discharged from the orthopedic clinic, I went to my surgeon’s office.

“I am sorry to interrupt. I just wanted to say thank you–“

“It’s fine, Magda. Your feet will be straight from now on.”

“Yes, but that’s not what I mean. I wanted to thank you for something else.”

“What is it?”

“No one will ever make fun of me again because of what you have done. Thank you for that.”

“No, Magda. No one will ever make fun of you, thanks to you.”

## Little Wonders

Marek Hłasko (2014), the Polish author and playwright, once wrote that everyone eventually comes back to the place they once wanted to run away from. Maybe this is why I decided to study nursing at the University of Medical Sciences in Poznań. I’m also a volunteer in the pediatric clinic where 21 years ago a couple of men in white coats saved my life. A year ago I had a chance to give a speech at an international HOPE congress (Picz, 2018) as a special guest, and now I’m writing my life’s story! If somebody had told me six years ago that I would be giving a speech in front of hundreds of people, I would have considered them insane, and yet here I am.

I would like to work with kids when I graduate. I’ve always liked children more than adults. They’re honest – they tell you the truth, no matter how horrible it may be. I would like to give them the unconditional kindness and help I have received from all the people who saved my life. I hope they know how grateful I am for them.

In my work, I see a lot of suffering. There are days when the pain seems unbearable, but on the other side of every horrible thing that happens, there is a small miracle. In fact, I see a lot of them every day. Little wonders are shining shyly behind the smiles of the patients, and that is more than enough to keep me going.

## Conclusion

Every single person is unique. I can only speak for myself, but I think that when children are seriously ill and go to the hospital, a part of the life they had been leading is taken away from them. Hospital teachers, occupational therapists, in fact, all medical staff are just the right people to bring a piece of that normality back. It is a tremendously significant ability, almost like a superpower.

There may be kids who can handle situations like this pretty well, but I am certain that among them there are some who feel lost, scared and alone. They don’t want sympathy. I didn’t want sympathy because it made me feel even more miserable than I was. At times when everything is new, the medical practitioners are like conductors of light. For me, not only did they bring back a little bit of my former reality when everything had been fine, but they also gave me something to talk about with friends – about school. I could now sort of identify with my friends, which made me feel much more confident.

I wish all medical practitioners reading this right now remember this. The extraordinary is in the simplest things we do every day, and this is why I call them small miracles. You are all irreplaceable. Never ever lose faith in what you do and always – *always* – remember why you started. You are not just doctors, nurses, teachers – you are life changers.

This is my story. You may ask who am I. I am everything that has happened to me, to my mom and to my whole family. I am made up of all those complicated emotions in the hearts of everyone I love. I am made of fear and sadness, excitement, hope and happiness, big mistakes and small miracles. This isn’t just my story and, therefore, my life isn’t just mine.

I am healthy and happy, hoping to be the reason that someone in the future will be happy and healthy, too.

## References

[1] Atkinson, R. (1998). The life story interview. Thousand Oaks, CA: SAGE Publications. DOI: 10.4135/9781412986205

[2] Hłasko, M. (2014). Sowa, coórka piekarza [Owl, the baker’s daughter]. Warszawa, Poland: Wydawn. Agora.

[3] Picz, M. (2018). Hospital education seen through the eyes of a patient. Presentation at 11th HOPE Congress, Poznań, Poland.

